# Identification of Novel Molecular and Clinical Biomarkers of Survival in Glioblastoma Multiforme Patients: A Study Based on The Cancer Genome Atlas Data

**DOI:** 10.1155/2024/5582424

**Published:** 2024-04-04

**Authors:** Luísa Esteves, Francisco Caramelo, Domingos Roda, Isabel Marques Carreira, Joana Barbosa Melo, Ilda Patrícia Ribeiro

**Affiliations:** ^1^Cytogenetics and Genomics Laboratory, Institute of Cellular and Molecular Biology, Faculty of Medicine, University of Coimbra, Coimbra, Portugal; ^2^University of Coimbra, Coimbra Institute for Clinical and Biomedical Research (iCBR) and Center of Investigation on Environment Genetics and Oncobiology (CIMAGO), Faculty of Medicine, Coimbra, Portugal; ^3^University of Coimbra, Center for Innovative Biomedicine and Biotechnology (CIBB) and Clinical Academic Center of Coimbra (CACC), Coimbra, Portugal; ^4^Laboratory of Biostatistics and Medical Informatics, iCBR-Faculty of Medicine, University of Coimbra, Coimbra, Portugal; ^5^Algarve Radiation Oncology Unit-Joaquim Chaves Saúde (JCS), Faro, Portugal

## Abstract

Glioblastoma multiforme (GBM) is the most prevalent type of brain tumour; although advancements in treatment have been made, the median survival time for GBM patients has persisted at 15 months. This study is aimed at investigating the genetic alterations and clinical features of GBM patients to find predictors of survival. GBM patients' methylation and gene expression data along with clinical information from TCGA were retrieved. The most overrepresented pathways were identified independently for each omics dataset. From the genes found in at least 30% of these pathways, one gene that was identified in both sets was further examined using the Kaplan-Meier method for survival analysis. Additionally, three groups of patients who started radio and chemotherapy at different times were identified, and the influence of these variations in treatment modality on patient survival was evaluated. Four pathways that seemed to negatively impact survival and two with the opposite effect were identified. The methylation status of *PRKCB* was highlighted as a potential novel biomarker for patient survival. The study also found that treatment with chemotherapy prior to radiotherapy can have a significant impact on patient survival, which could lead to improvements in clinical management and therapeutic approaches for GBM patients.

## 1. Introduction

Gliomas are the most common presentation of malignant primary brain tumours worldwide, and among those, 54.4% are classified as glioblastoma multiforme (GBM). Patients are usually diagnosed at an average age of 64, and men are about 1.5 times more likely to develop GBM than women [1]. As to the aetiology of the disease, known risk factors are linked to exposure to therapeutic ionizing radiation and some chemicals, like some substances derived from the petroleum refinery industry and tobacco. Around 5% of all GBM cases are also associated with hereditary syndromes [1].

Worldwide, GBM has an incidence of 3.9 cases per 100,000-person years, and most patients exhibit a poor prognosis, with only 2% of patients surviving three years or longer after diagnosis [2–4]. A multimodal treatment known as the Stupp protocol is the norm, with maximum tumour resection followed by a treatment course of radiotherapy with concomitant chemotherapy with temozolomide followed by additional adjuvant cycles of chemotherapy [5]. Despite the aggressiveness of the most widely used treatment approaches and advancements in the research of novel treatment modalities, survival of GBM patients has had little to no improvement in the last decades, with the median survival time being around 14.6 months and most patients ending up with early disease progression or disease recurrence [1, 4].

The research of molecular markers that can lend both diagnostic and prognostic insights to the clinical presentation of GBM is ever so important. Some biomarkers have become the hallmark of tumour assessment, and, in some subtypes of gliomas, they have been shown to be helpful in guiding the clinical management of the patients. For example, the methylation status of the O-6-methylguanine-DNA methyltransferase (MGMT) promoter was clinically shown to be a strong predictive biomarker for temozolomide sensitivity [6, 7]. *IDH* mutation has emerged as a marker of better prognosis in low- and high-grade gliomas, in adults, and a mutation in *EGFR*, EGFRvIII, has also been studied as a biomarker related to tumour response and relapse as well as a potential therapeutic target [6]. Alteration and/or increased activity in the Wnt, transforming growth factor *β* (TGF-*β*), VEGF, epidermal growth factor receptor (EGFR), cyclin-dependent kinase 2A (CDKN2A), nuclear factor-*κ*B (NF-*κ*B), and phosphatidylinositol-3-kinase (PI3K)/AKT/mammalian target of rapamycin (mTOR) pathways may be linked to the GBM pathogenesis and aggressive tumour behaviour [8].

Even with significant developments in bioinformatics pipelines [9, 10] and molecular techniques, the exact ways in which GBM develops and progresses are not yet completely understood. It is important to stress that the pathophysiology of GBM appears to depend on a wide range of oncogenes or tumour suppressor genes, hindering the development of drug efficacy targeting only one molecular alteration in clinical trials [11]. It is fundamental to identify the main aberrant molecular targets and pathways in the onset and progression of GBM in order to understand the pathology of GBM and to create new therapeutic approaches.

Recent research has shown that the progression of glioma is connected to various types of epigenetic changes, such as changes to histones, DNA methylation, and chromatin structure, as well as abnormal microRNAs. The genes and proteins that regulate these changes have become potential targets for novel treatments [12]. Changes in gene expression have also been shown to lead to tumour progression [13].

Genomic and epigenetic characterization of GBM has allowed the refinement of the classification of these tumours; however, effective treatment options remain very limited. Several factors seem to be responsible for the failure of treatment, such as the blood-brain barrier (BBB), which restricts the entry of chemotherapeutic agents into the tumour; the intratumoral presence of cancer stem cells that are chemo- and radioresistant; and the infiltrative nature of GBM cells that hampers complete surgical resection [14, 15].

Novel molecular biomarker discovery can be decisive in influencing clinical outcomes, especially in it comes to the survival of the patients, which, in the case of GBM, is remarkably low. In this study, we analysed methylation and gene expression profiles of GBM patients to identify potential molecular and clinical markers of survival to help elucidate the pathogenicity of this kind of neoplasm. By applying various statistical methodologies, we identified several factors, for instance, certain signalling pathway, the methylation profile of *PRKCB*, and the treatment modality where chemotherapy is started prior to radiotherapy, that can all have an impact on patient survival and influence decision-making in the clinical practice.

## 2. Materials and Methods

### 2.1. Methylation and Gene Expression Data Reduction

Methylation, gene expression, and clinical data from 573 GBM patients were retrieved from The Cancer Genome Atlas. Considering methylation data, a hard threshold value of 0.25 methylation level was set, and only genes that were altered in at least 30% of patients were kept for further analysis. mRNA expression data were filtered by removing genes with over 50% of null values, and *z*-scores were calculated for each gene. A gene was only considered to be altered if the *z*-score was lower than -1.96 or higher than 1.96, and only genes that were altered in, at least, 70% of patients were kept. Various methylation cut-off values (0.20–0.30) were evaluated, yielding minimal variance in the outcomes. In contrast, the selected *z*-score values (-1.96 and 1.96) are associated with the definition of the 95% confidence interval. This signifies that the likelihood of encountering values exceeding the absolute value of the cut-off is 5%, which, in general, is not considered normal.

### 2.2. Signalling Pathway and Survival Analysis

After the data reduction step described before, signalling pathway analysis was performed using *limma* from Bioconductor. The signalling pathway analysis was done separately for methylation and expression, resulting in the most overrepresented pathways (*p* < 0.05) in both omics (expression and methylation). Pathways were sorted in ascending order by *p* values, and only the top ten signalling pathways from each of the omics, methylation and expression, were selected based on the smallest *p* values to evaluate their association with the survival of the patients aiming to establish a prognosis profile for GBM patients.

For each patient, the percentage of genes that were altered from a given pathway was calculated. These percentages were then used as independent variables in a Cox regression model, with the aim of determining which pathways contribute most to the survival rate by analysing the model coefficients that represent hazard ratios. The proportional hazards assumption was verified by visual inspection of the graphs of the Schoenfeld residuals over time.

In order to further explore the genetic makeup of GBM and its association with the patient's survival, the genes present in, at least, 30% of the most overrepresented pathways were selected, which resulted in two sets of seven genes, one for each of the omics. While this approach was designed with the intention of capturing the most influential genes contributing to various pathways, it is important to acknowledge certain limitations inherent in studying only a single gene combination.

One gene was common in both sets, which prompted us to further investigate it, finding that the gene was predominantly underexpressed (in over 90% of samples). To determine how this gene could impact on survival, a Kaplan-Meier analysis based only on the methylation status of this gene was performed. The expression profile was not used as it was fairly homogenous, since more than 90% of subjects presented the gene underexpressed, and thus no distinguishable and balanced groups could be established.

### 2.3. Evaluation of the Influence of Treatment Regimens on the Survival of the Patients

All 319 patients with documented treatment details underwent a standardized glioblastoma treatment regimen, namely, the Stupp protocol, which involves the concurrent administration of radiation and chemotherapy [5]. Within the available clinical data, the patient cohort was stratified into three distinct groups based on the initiation timing of their treatments. The first group comprised 176 patients who commenced both radiation and chemotherapy either on the same day or within a thirty-day interval (hereafter denoted as Group 1). A second group consisted of 69 patients who initiated radiation treatment first (Group 2), with chemotherapy administered more than thirty days later. Additionally, a third group encompassed 74 patients (Group 3) who commenced chemotherapy more than thirty days before initiating radiotherapy. These delineations, elucidating the temporal initiation patterns of treatment, provide a comprehensive framework for characterizing the patient cohorts under consideration. The frequency of these groups was evaluated in the group that had higher survival rates in the previous analysis, and the influence of the treatment regimen on survival was evaluated in the cohort through the Kaplan-Meier method.

## 3. Results

### 3.1. Signalling Pathway and Survival Analysis

A total of 61 overrepresented signalling pathways were identified for the methylation dataset. As for the gene expression data, 104 pathways were considered significant. The top ten signalling pathways for each of the omics are represented in Supplementary Materials Figures 1 and 2.

Cox regression was performed on both sets of signalling pathways, with the results being shown in Tables 1 and 2.

For the methylation dataset (Table 1), the neuroactive ligand-receptor interaction, cytokine-cytokine receptor interaction, amoebiasis, and cAMP signalling pathways all showed statistical significance in contributing to the survival of the patients.

In the expression data (Table 2), no relation was found between the percentage of altered genes in a given pathway and the risk of death in GBM patients. Although the serotonergic synapse pathway showed marginal significance, the 95% confidence interval crosses over 1, meaning that there is no impact on the survival of the patients.

**Figure 1:**
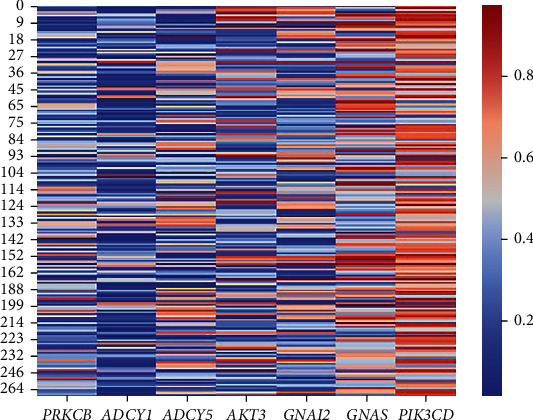
Heatmap representing the methylation values of the seven genes present in, at least, 30% of the most overrepresented pathways in the methylation dataset (*N* = 283). Represented in the columns are the methylation values related to each gene and the rows represent each patient.

**Figure 2:**
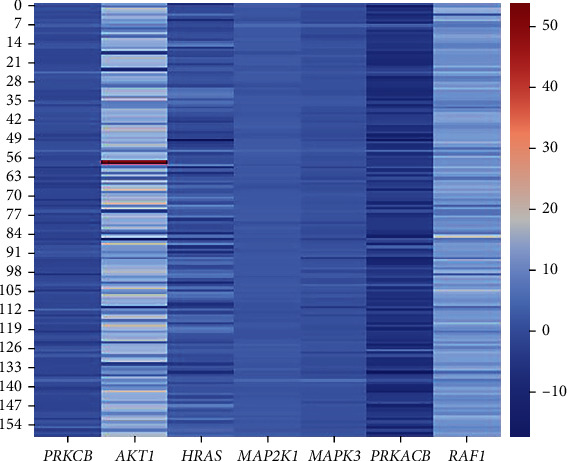
Heatmap representing the expression values of the seven genes present in, at least, 30% of the most overrepresented pathways in the gene expression dataset (*N* = 159). Represented in the columns are the methylation values related to each gene and the rows represent each patient.

To further characterize the genetic alterations that occur in GBM and their association with the patient's survival, we considered the genes present in over 30% of the most overrepresented pathways resulting in two sets of seven genes, one for each of the omics. Both sets of genes are represented in Figures 1 and 2.

It can be observed that genes pertaining to the methylation data (Figure 1) show a wide range of values and that those from the expression dataset (Figure 2) show underexpression in the majority of the patients.

*PRKCB* was found in both sets of genes, making it a good target for further investigation as a potential biomarker in GBM. However, since the expression values did not allow for the observation of distinct groups, only the relationship between the methylation status of this gene and survival was evaluated.

**Figure 3:**
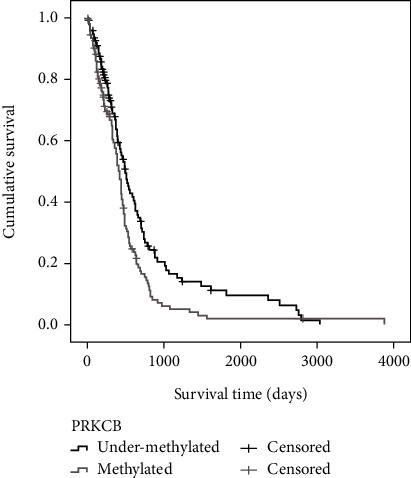
Kaplan-Meier survival curves for the PRKCB methylation status (undermethylated and methylated). Survival time is presented in days. Patients with a *PRKCB* undermethylated profile survived 79 days longer in median survival time (*p* < 0.01).

Survival analysis using the Kaplan-Meier method based on the two different methylation statuses of this gene was performed (Figure 3). From there, it was determined that there were two distinct survival groups established by the methylation status of *PRKCB*. The different methylation *PRKCB* profiles exhibited a difference of 79 days (2.6 months) in median survival time (*p* < 0.01), with those that were undermethylated performing better (Table 3).

The impact of the other genes on survival was also evaluated; however, no significant effect was observed in this cohort (results not shown).

### 3.2. Evaluation of the Influence of Treatment Regimens on the Survival of the Patients

Since the group of patients with undermethylated *PRKCB* showed higher survival rates, we wanted to evaluate if the treatment regimen could create a bias in the survival of the patients. After performing Fisher's exact test, the treatment modality did not seem to have a statistically significant association with the survival groups (*p* = 0.07). However, different treatment regimens do exist in the cohort and, as an important factor to consider for patient survival, we wanted to evaluate if patients survived differently according to the treatment they were exposed to. In total, 70% of patients with available information started both treatments at the same time or within a 30-day interval (Group 1), 12% started radiation more than thirty days prior to starting chemotherapy (Group 2), and 18% started chemotherapy more than thirty days after radiotherapy (Group 3).

Kaplan-Meier curves were plotted for the three groups, and through the Log-Rank test, it was determined that there were differences between the groups in terms of survival (*p* = 0.04). In order to determine if the observable differences were being influenced by the staging of the patients, Cramer's *V* was calculated for the Karnofsy performance status of the patients, by group. This value was 0.033, which according to Cohen's rule (1988) indicates a very small effect size, meaning that Karnofsy's index is not differentially distributed across groups and differences in survival cannot be attributed to the staging of the patients.

**Figure 4:**
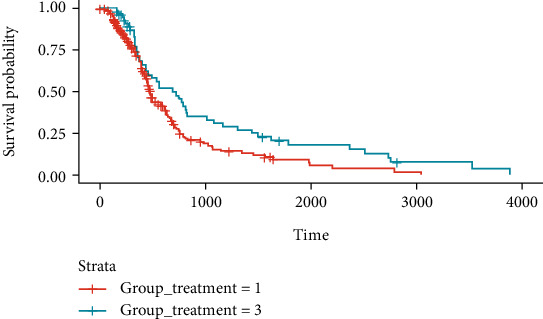
Kaplan-Meier survival curves for treatment Groups 1 (in salmon) and 3 (in turquoise). Survival time is presented in days. Group 3 patients survived 215 days longer than patients in Group 1 (*p* = 0.01).

Knowing that there were significant differences between the treatment groups, we wanted to establish between which groups they were detectable. In order to achieve this, the groups were compared two by two using the Kaplan-Meier method. We were able to determine that Groups 1 and 3 had statistically significant differences in what comes to survival (*p* = 0.01) in which patients in Group 3 survived, in median, 215 days (approximately 7 months) longer than those in Group 1 (Figure 4).

## 4. Discussion

The study of GBM is an ever-evolving and progressing field of research, in the molecular characterization of this type of carcinoma as well as the investigation of more effective treatment options [1]. Some known mechanisms contribute to the resistance to treatment that these tumours present, such as the preferential activation of DNA-damage response pathways in glioma stem cells in response to radiotherapy and the inhibition of apoptosis, upregulation of multidrug resistance genes and the genomic rearrangement of *MGMT* contribute to the resistance to standard chemotherapy with temozolomide [1]. GBM remains one of the deadliest human neoplasms, with modest improvements in overall survival in the long run [1]. Thus, the main objective of this study was to research the expression and methylation profiles along with the clinical data of GBM patients aiming to uncover a more comprehensive picture of this neoplasm's pathogenesis and, consequently, to identify potential predictors of patient survival.

We were able to identify a total of 61 and 104 signalling pathways overrepresented in the methylation and gene expression datasets, respectively. They were majorly related to carcinogenic processes, cell cycle progression, and neurobiological processes. The most overrepresented pathway was the neuroactive ligand-receptor interaction pathway in both datasets. Also, common to both groups were the cytokine-cytokine interaction, serotonergic synapse, viral protein interaction with cytokine and cytokine receptor, hematopoietic cell line, amoebiasis, legionellosis, and Staphylococcus aureus infection pathways. Additionally, some of these pathways have already been associated with GBM in some prognostic studies [16–18]. It is important to emphasised that GBM is molecularly very heterogeneous, being identified through genomic analysis, several signalling pathways, and gene alterations that are critical for its development [8]. A TCGA study analysed the mutational landscape in GBM, identifying the three main genetic events: amplification and mutational activation of RTK genes, activation of the PI3K pathway, and inactivation of the p53 and retinoblastoma tumour suppressor pathways [19]. According to Cox regression, for the methylation dataset, neuroactive ligand-receptor interaction and amoebiasis pathways were statistically significant for the survival of the patients. An increase of one unit in the percentage of altered genes from the neuroactive ligand-receptor interaction and amoebiasis pathways corresponds to an increase in the risk of death on average of 1.032 and 1.026 times, respectively. Age also shows statistical significance, and for each year older, the risk of dying increases by 1.040 times.

From the two sets of genes present in the majority of identified pathways, *PRKCB* is worthy of notice. Different methylation *PRKCB* profiles exhibited a difference of 2.6 months in median survival time (*p* < 0.01) which represents a difference of around 15% between median survival times, with patients that presented the undermethylated gene surviving longer. *PRKCB* is involved in pathways related to neurological function and carcinogenic processes, including the glioma, MAPK, and Rap1 signalling pathways. *PRKCB* has also been shown to participate in the regulation of the rate of autophagy, which can either promote cell death or activate prosurvival mechanisms [20]. The increased expression of *PRKCB* is seen as advantageous and has been linked to improved relapse-free survival for individuals with breast cancer [21]. *PRKCB* promoter methylation has been associated with prostate cancer, being able to independently predict disease recurrence, and with non-small cell lung cancer, where the level of *PRKCB* promoter methylation was notably greater in tumour tissue in comparison to the surrounding tissue, making *PRKCB* a potential methylation biomarker for the diagnosis of that type of cancer [22, 23]. *PRKCB* belongs to the protein kinase C (PKC) family. PKC isoforms have tumour-promoting properties, acting as enhancers to multiple cellular signalling pathways. This family is also involved in the regulation of cell survival and apoptosis [24].

Some predictive studies have identified *PRKCB* expression profiles as part of signatures related to survival [25, 26]. However, to the best of our knowledge, this is the first study to report the methylation profile of *PRKCB* as a potential prognostic factor. Since the overall survival of GBM patients has been persistent at around 15 months in median, a survival of 2.6 months longer that might seem small for other pathologies is a tangible significant improvement for GBM. Validation studies in larger cohorts are needed to definitively establish this prognostic biomarker.

Regarding treatment, the standard procedure consists of maximal tumour resection followed by six weeks of radiotherapy with concurrent chemotherapy, most commonly, temozolomide, with additional adjuvant cycles of chemotherapy. The simultaneous administration of radio and chemotherapy has resulted in a median overall survival of 14.6 months and a survival rate of 26.5% after two years, in opposition to the 10.4% two-year survival rate of patients treated with radiotherapy alone [1, 5]. In TCGA cohort, we found that there were three groups of patients that, even though were exposed to multimodal therapy, started the two modalities of treatment at different times. We were able to determine that those patients who were treated with chemotherapy followed by radiotherapy after more than thirty days survived, in median, 7 months longer than those that started both treatments at once or within a thirty-day interval. This discovery could have a real impact on the standard of care, seeing that patients who were treated in a nonconventional manner seemed to survive longer. We were unable to find any studies that have taken the order of treatments into account, so we believe that this is the first study that identifies an improvement in survival that comes with starting chemotherapy before radiation. Furthermore, a randomized clinical trial performed between 2005 and 2009 in patients with astrocytoma or glioblastoma, aiming to compare the efficacy of radiotherapy alone versus temozolomide alone, found no clear advantage of one over the other, and since the establishment of the Stupp protocol allowed for the improvement of survival in GBM patients, the administration of the two forms of treatment seems to be the best option [5, 27]. Nevertheless, following our findings, we believe that further studies should be conducted in other independent cohorts in order to evaluate the effect of chemotherapy given more than a month prior to radiotherapy.

However, it should be noted that these findings are due to the available information relating to the treatment courses and the treatment plans could have been changed for certain patients due to time or logistic constraints of the hospitals where they were treated.

We can speculate that the decision of when to start radiotherapy or chemotherapy can be influenced by a variety of factors. These may include the specific policies and practices of the medical institution providing the treatment, the availability of equipment and resources, and the patient's health and recovery following surgery (potential side effects, infection, and other comorbidities). Additionally, there may be delays in starting radiotherapy due to economic considerations, such as the need to obtain authorization from health insurance companies. In some cases, technical issues or maintenance of the radiotherapy equipment may also impact the timing of treatment, justifying starting chemotherapy first. Furthermore, some services might have administered previous treatment plans up until the validation and widespread practice of the Stupp protocol, delaying its full acceptance. However, these involuntary findings, once grouped into specific time intervals, may translate into a possible enhancement of tumour control after surgery. Premature introduction of chemotherapy can, theoretically, promote early control of residual microscopic disease at the surgical bed.

As such, the standards of care may not have been followed for practical reasons, and more studies related to this topic should be performed.

There were some potential limitations to this observational single cohort study mainly due to the lack of available clinical information, which mostly hindered our efforts to correlate the genetic and epigenetic information with the clinical data and limited the possibilities of understanding the relationship between the clinical data and the survival of the patients. The extent of surgical resection as well as the site of the tumour has been proven to be associated with prognosis [28]; however, incomplete information on the database prevented further studies on this matter. Furthermore, the survival of the patients is characteristically low, and as such, the follow-up period of the patients is low. To become clinically useful, these factors need systematic validation in larger patient cohorts from well-designed clinical trials to increase specificity and sensitivity and be able to set solid threshold values for predicting tumour response to treatments and survival.

## 5. Conclusions

In conclusion, we report several potential new indicators of patient survival. Firstly, we identified four signalling pathways that seem to negatively impact survival as well as two others that could diminish the risk of death, given the percentage of genes altered by the pathway. Additionally, the methylation status of *PRKCB* was revealed as a potential novel biomarker for patient survival, and lastly, we found that the treatment with chemotherapeutic agents prior to radiotherapy can have a significant impact on the survival of the patients, which could lead to major improvements in clinical management and therapeutic approaches for GBM patients. It is important to stress that this study showed the importance of different omics analyses to perform a comprehensive characterization of GBM and consequently to identify biomarkers with clinical utility, opening new doors for further research. Our results represent a step forward to improve patients' management and eventually to guide new therapeutic target development. Considering that overall survival of GBM patients keeps around 15 months in median, an improvement of 2.6 months, as we identified in this study when the *PRKCB* gene is not methylated, could represent an important impact on the GBM patient's management and in the clinical practice. Further multicentric studies should be conducted in independent cohorts in order to allow the generalization and validation of these findings.

## Figures and Tables

**Table 1:** Cox regression performed on the top ten signalling pathways determined for the methylation data.

Signalling pathway	*B*	SE	*p* value	HR	95% CI for HR	
					Lower	Upper
Neuroactive ligand-receptor interaction	0.031	0.014	0.029	1.032	1.003	1.061
Staphylococcus aureus infection	0.003	0.011	0.799	1.003	0.981	1.026
Cytokine-cytokine receptor interaction	-0.033	0.017	0.055	0.968	0.936	1.001
Hematopoietic cell lineage	0.022	0.011	0.057	1.022	0.999	1.045
Complement and coagulation cascades	-0.008	0.010	0.413	0.992	0.973	1.011
Viral protein interaction with cytokine and cytokine receptor	0.004	0.015	0.792	1.004	0.975	1.033
Legionellosis	-0.002	0.007	0.777	0.998	0.984	1.012
Amoebiasis	0.025	0.011	0.016	1.026	1.005	1.047
Serotonergic synapse	-0.014	0.009	0.122	0.986	0.968	1.004
cAMP signalling pathway	-0.023	0.012	0.061	0.977	0.954	1.001
Age (years)	0.039	0.006	<0.001	1.040	1.027	1.053

**Table 2:** Cox regression performed on the top ten signalling pathways determined for the expression data.

Signalling pathway	*B*	SE	*p* value	HR	95% CI for HR	
					Lower	Upper
Neuroactive ligand-receptor interaction	-0.007	0.025	0.792	0.993	0.946	1.043
Staphylococcus aureus infection	0.000	0.022	0.995	1.000	0.958	1.043
Cytokine-cytokine receptor interaction	-0.013	0.028	0.640	0.987	0.934	1.043
Hematopoietic cell lineage	0.013	0.022	0.558	1.013	0.971	1.057
Complement and coagulation cascades	0.023	0.018	0.206	1.023	0.988	1.060
Viral protein interaction with cytokine and cytokine receptor	0.002	0.018	0.907	1.002	0.967	1.038
Legionellosis	-0.023	0.018	0.208	0.977	0.943	1.013
Amoebiasis	0.014	0.020	0.477	1.015	0.975	1.056
Serotonergic synapse	-0.048	0.026	0.067	0.953	0.905	1.003
cAMP signalling pathway	0.034	0.030	0.257	1.034	0.976	1.096
	0.039	0.010	<0.001	1.040	1.019	1.060

**Table 3:** Means and medians for survival times, determined by the Kaplan-Meier method, for the two PRKCB methylation statuses. Survival is shown in days.

*PRKCB* methylation status	Mean				Median			
	Estimate	SE	95% CI		Estimate	SE	95% CI	
			Lower	Upper			Lower	Upper
Undermethylated	741.3	78.7	587	895.6	498	41.8	416.2	579.8
Methylated	501.7	56.5	390.9	612.6	419	24.7	370.6	467.4
Overall	611.6	48.3	516.8	706.4	438	25.1	388.7	487.3

## Data Availability

The data used to support the findings of this study was downloaded from The Cancer Genome Atlas (TCGA) (https://portal.gdc.cancer.gov/).

## Supplementary Materials

Supplementary Materials Supplementary Figure 1: Sankey diagram representing the top ten overrepresented signalling pathways for the methylation dataset. Supplementary Figure 2: Sankey diagram representing the top ten overrepresented signalling pathways for the gene expression dataset.

## References

[1] Alfieries C., Trafalis D. (2015). Glioblastoma multiforme: pathogenesis and treatment. *Pharmacology & Therapeutics*.

[2] Batash R., Asna N., Schaffer P., Francis N., Schaffer M. (2017). Glioblastoma multiforme, diagnosis and treatment; recent literature review. *Current Medicinal chemistry.*.

[3] Carlsson S. K., Brothers S. P., Wahlestedt C. (2014). Emerging treatment strategies for glioblastoma multiforme. *EMBO Molecular Medicine*.

[4] Tykocki T., Eltayeb M. (2018). Ten-year survival in glioblastoma. A systematic review. *Journal of Clinical Neuroscience*.

[5] Stupp R., Mason W. P., van den Bent M. J. (2005). Radiotherapy plus concomitant and adjuvant temozolomide for glioblastoma. *The New England Journal of Medicine*.

[6] Siegal T. (2016). Clinical relevance of prognostic and predictive molecular markers in gliomas. *Advances and Technical Standards in Neurosurgery*.

[7] Sasmita A. O., Wong Y. P., Ling A. P. K. (2018). Biomarkers and therapeutic advances in glioblastoma multiforme. *Asia-Pacific Journal of Clinical Oncology*.

[8] Khabibov M., Garifullin A., Boumber Y. (2022). Signaling pathways and therapeutic approaches in glioblastoma multiforme. *International Journal of Oncology.*.

[9] Dang H. H., Ta H. D. K., Nguyen T. T. T. (2022). Prospective role and immunotherapeutic targets of sideroflexin protein family in lung adenocarcinoma: evidence from bioinformatics validation. *Functional & Integrative Genomics*.

[10] Tran TO, Vo T. H., Lam L. H. T., Le N. Q. K. (2023). ALDH2 as a potential stem cell-related biomarker in lung adenocarcinoma: comprehensive multi-omics analysis. *Computational and Structural Biotechnology Journal*.

[11] Rajesh Y., Pal I., Banik P. (2017). Insights into molecular therapy of glioma: current challenges and next generation blueprint. *Acta Pharmacologica Sinica*.

[12] Uddin S., Mamun A. A., Alghamdi B. S. (2022). Epigenetics of glioblastoma multiforme: from molecular mechanisms to therapeutic approaches. *Seminars in Cancer Biology*.

[13] Qiang L., Aishwarya S., Ji-Ping L., Dong-Xiao P., Jia-Pei S. (2022). Gene expression profiling of glioblastoma to recognize potential biomarker candidates. *Frontiers in Genetics*.

[14] Zong H., Parada L. F., Baker S. J. (2015). Cell of origin for malignant gliomas and its implication in therapeutic development. *Cold Spring Harbor Perspectives in Biology*.

[15] Khaddour K., Johanns T. M., Ansstas G. (2020). The landscape of novel therapeutics and challenges in glioblastoma multiforme: contemporary state and future directions. *Pharmaceuticals*.

[16] Wang J. J., Wang H., Zhu B. L. (2021). Development of a prognostic model of glioma based on immune-related genes. *Oncology Letters*.

[17] Gao W., Li Y., Zhang T. (2022). Systematic analysis of chemokines reveals CCL18 is a prognostic biomarker in glioblastoma. *Journal of Inflammation Research*.

[18] Ye S., Yang B., Zhang T. (2022). Identification of an immune-related prognostic signature for glioblastoma by comprehensive bioinformatics and experimental analyses. *Cells*.

[19] The Cancer Genome Atlas Research Network (2008). Comprehensive genomic characterization defines human glioblastoma genes and core pathways. *Nature*.

[20] Wang K., Klionsky D. J. (2011). Mitochondria removal by autophagy. *Autophagy*.

[21] Roessler J., Ammerpohl O., Gutwein J. (2015). The CpG island methylator phenotype in breast cancer is associated with the lobular subtype. *Epigenomics*.

[22] Daniunaite K., Bakavicius A., Zukauskaite K. (2021). Promoter methylation of PRKCB, ADAMTS12, and NAALAD2 is specific to prostate cancer and predicts biochemical disease recurrence. *International Journal of Molecular Sciences*.

[23] Liu S., Chen X., Chen R. (2017). Diagnostic role of Wnt pathway gene promoter methylation in non-small cell lung cancer. *Oncotarget*.

[24] Martiny-Baron G., Fabbro D. (2007). Classical PKC isoforms in cancer. *Pharmacological Research*.

[25] Serão N. V., Delfino K. R., Southey B. R., Beever J. E., Rodriguez-Zas S. L. (2011). Cell cycle and aging, morphogenesis, and response to stimuli genes are individualized biomarkers of glioblastoma progression and survival. *BMC Medical Genomics*.

[26] Zhang Y., Xu J., Zhu X. (2018). A 63 signature genes prediction system is effective for glioblastoma prognosis. *International Journal of Molecular Medicine*.

[27] Wick W., Platten M., Meisner C. Temozolomide chemotherapy alone versus radiotherapy alone for malignant astrocytoma in the elderly: the NOA-08 randomised, phase 3 trial. http://www.thelancet.com/oncology.

[28] Armocida D., Pesce A., Palmieri M. (2021). Periventricular zone involvement as a predictor of survival in glioblastoma patients: a single centre cohort-comparison investigation concerning a distinct clinical entity. *Neurosurgery*.

