# Supplementary material for: Identification of Novel Molecular and Clinical Biomarkers of Survival in Glioblastoma Multiforme Patients: A Study Based on The Cancer Genome Atlas Data
Source: Biomed Res Int. 2024 Apr 4;2024:5582424. doi: 10.1155/2024/5582424 (PMC11008977; doi:10.1155/2024/5582424)
Supplement: Supplementary Materials — Supplementary Figure 1: Sankey diagram representing the top ten overrepresented signalling pathways for the methylation dataset. Supplementary Figure 2: Sankey diagram representing the top ten overrepresented signalling pathways for the gene expression dataset. [file 5582424.f1.docx]

**Supplementary Material**

**
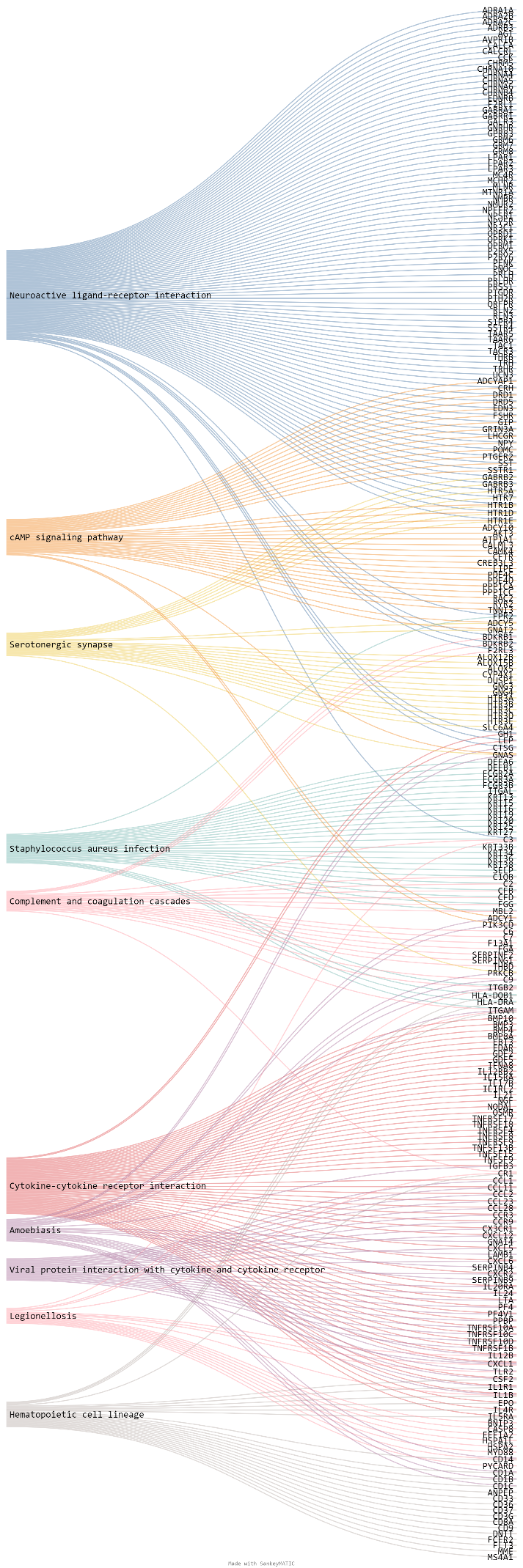
**

Supplementary Figure 1 - Sankey Diagram representing the top ten overrepresented signalling pathways for the methylation data set

**
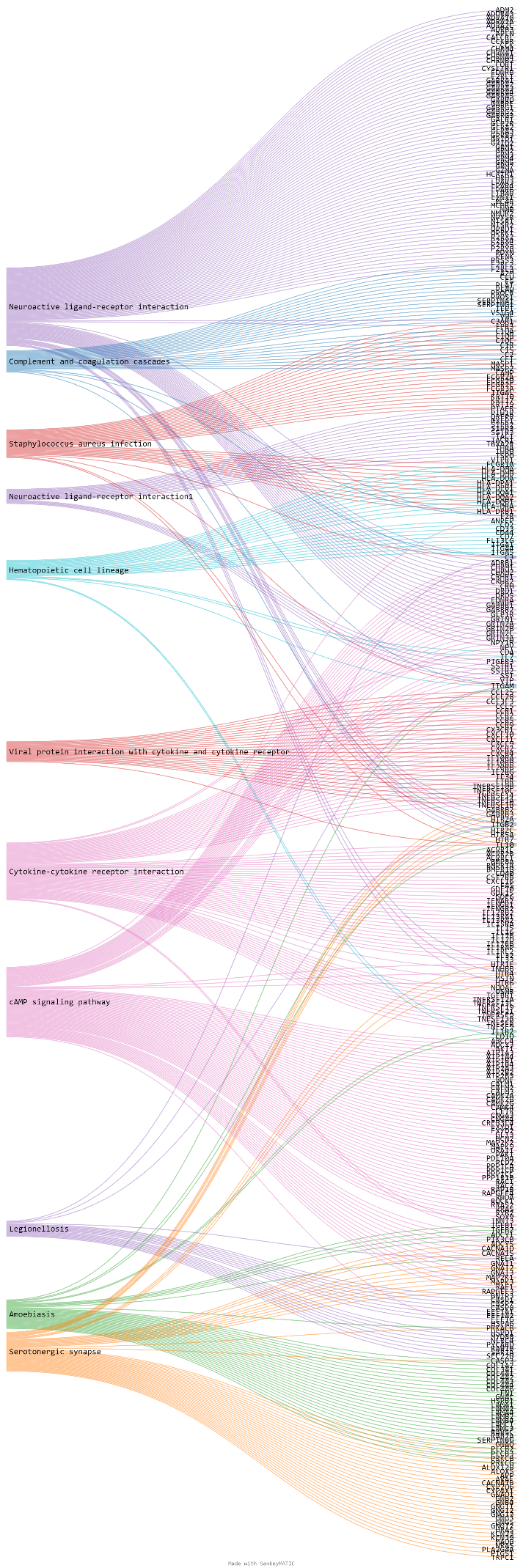
**

Supplementary Figure 2 - Sankey Diagram representing the top ten overrepresented signalling pathways for the gene expression data set.
